# Supplementary material for: Chaos controlled and disorder driven phase transitions induced by breaking permutation symmetry
Source: Sci Rep. 2025 Oct 7;15:34984. doi: 10.1038/s41598-025-18895-x (PMC12504712; doi:10.1038/s41598-025-18895-x)
Supplement: Supplementary file 1 — Supplementary Information. [file 41598_2025_18895_MOESM1_ESM.pdf]

Chaos controlled and disorder driven phase transitions induced by breaking permutation symmetry

Manju C[1], Arul Lakshminarayan[2], Uma Divakaran[3,*]

[1]Department of Physics, Indian Institute of Technology Palakkad, Kerala, India 678623
[2]Department of Physics, & Center for Quantum Information, Computation and Communication, Indian Institute of Technology Madras, Chennai, India 600036
[3]Department of Physics, Indian Institute of Technology Palakkad, Kerala, India 678623

[*]Corresponding author: uma@iitpkd.ac.in

1 Solvable case of disordered Hamiltonian without kicks

The Hamiltonian without kicks is given by:

$$H = \frac{k}{2N} \sum_{\ell<\ell'=1}^{N} (1+\varepsilon_{\ell\ell'})\sigma_\ell^x \sigma_{\ell'}^x \,. \tag{1}$$

We need to find the expectation value of J^2 at time t when the initial state is the spin coherent state

$$|\psi_0\rangle = \left(\cos\frac{\theta}{2}\,|0\rangle + e^{i\phi}\sin\frac{\theta}{2}\,|1\rangle\right)^{\otimes N}. \tag{2}$$

Since $J_x^2 = \left(\sum_{\ell=1}^N \sigma_\ell^x\right)^2/4$ commutes with the Hamiltonian, $\left\langle J_x^2(t)\right\rangle = \langle\psi_t|J_x^2|\psi_t\rangle = \langle\psi_0|J_x^2|\psi_0\rangle$. Hence, noting that the single qubit expectation of σ_x in the initial spin coherent state $|\psi_0\rangle$ is $\langle\sigma_x\rangle = \sin\theta\cos\phi$, we get

$$\left\langle J_x^2(t)\right\rangle = \frac{N}{4} + \frac{N(N-1)}{4}\sin^2\theta\cos^2\phi. \tag{3}$$

Similarly, writing the collective angular momentum operators $J_{y,z}$ in terms of Pauli $\sigma_{y,z}$

$$J_y^2 + J_z^2 = \frac{N}{2}\mathbb{I}_{2^N} + \frac{1}{2}\sum_{i<j}(\sigma_i^y\sigma_j^y + \sigma_i^z\sigma_j^z). \tag{4}$$

We first express the initial state $|\psi_0\rangle$ in the σ_x basis as:

$$|\psi_0\rangle = \frac{1}{2^{N/2}} \sum_{s_1,\cdots,s_N\in\{+,-\}} \alpha_+^{n_+(s)}\alpha_-^{n_-(s)}\,|s_1,\cdots,s_N\rangle \tag{5}$$

where $\alpha_\pm = (\cos\frac{\theta}{2} \pm e^{i\phi}\sin\frac{\theta}{2})$, $n_+(s)$ is the no of $+$ spins in the configuration $(s_1,s_2,\cdots s_N)$ and $n_-(s) = N - n_+(s)$. The evolved state at time t is $|\psi_t\rangle = U^t\,|\psi_0\rangle$ where $U = \exp\left(-\frac{ik}{2N}\sum_{l<l'=1}^N (1+\varepsilon_{ll'})\sigma_l^x\sigma_{l'}^x\right)$

The general form of evolved state $|\psi_t\rangle$ in full 2^N basis states is given by:

$$|\psi_t\rangle = \sum_{s_1,s_2,\cdots s_N\in\{+,-\}} c_{s_1,s_2\cdots s_N}(t)\,|s_1,s_2,\cdots s_N\rangle\,, \tag{6}$$

with

$$c_{s_1,s_2,\cdots s_N}(t) = \frac{1}{2^{N/2}}(\alpha_+)^{n_+(s)}(\alpha_-)^{n_-(s)}\exp\left(-\frac{ikt}{2N}\sum_{l<l'}(1+\varepsilon_{ll'})(2\delta_{s_l s_{l'}}-1)\right). \tag{7}$$

To calculate the expectation value of $\frac{1}{2}\sum_{i<j}(\sigma_i^y\sigma_j^y + \sigma_i^z\sigma_j^z)$ in state $|\psi_t\rangle$, denoted as $\langle O\rangle$, we first look at the action of single term in the summation, which is given by,

$$\frac{1}{2}(\sigma_i^y\sigma_j^y + \sigma_i^z\sigma_j^z)\,|s_1,s_2,\cdots s_i,\cdots s_j,\cdots s_N\rangle = \begin{cases} 0 & \text{if } s_i = s_j \\ |s_1,s_2,\cdots \bar{s}_i,\cdots \bar{s}_j,\cdots s_N\rangle & \text{if } s_i \neq s_j \end{cases} \tag{8}$$

where $\bar{s}$ is the flipped spin. Note that this implies that

$$\frac{1}{2}\sum_{i<j}(\sigma_i^y\sigma_j^y+\sigma_i^z\sigma_j^z)\,|\psi_t\rangle=\sum_{i<j}\sum_{\substack{s_1,s_2,\cdots s_N\in\{+,-\}\\ s_i\neq s_j}} c_{s_1,s_2,\cdots s_i\cdots s_j\cdots s_N}(t)\,|s_1,s_2,\bar{s}_i,\cdots\bar{s}_j,\cdots s_N\rangle\,. \tag{9}$$

Taking overlap with $\langle\psi_t|$ and then evaluating the coefficients gives:

$$\begin{aligned}\langle O\rangle &= \sum_{i<j}\sum_{\substack{s_1,s_2,\cdots s_N\in\{+,-\}\\ s_i\neq s_j}} c^*_{s_1,s_2,\cdots\bar{s}_i\cdots\bar{s}_j\cdots s_N}(t)\,c_{s_1,s_2,\cdots s_i\cdots s_j\cdots s_N}(t)\\ &= \frac{1}{2^N}\sum_{i<j}\sum_{\substack{s_1,s_2,\cdots s_N\in\{+,-\}\\ s_i\neq s_j}} \alpha_+^{n_+(s)}\alpha_-^{n_-(s)}(\alpha_+^*)^{n_+(s)}(\alpha_-^*)^{n_-(s)}\exp\left(-\frac{ikt}{2N}\sum_{l<l'}(1+\varepsilon_{ll'})(2\delta_{s_ls_{l'}}-1)\right)\\ &\quad\times\exp\left(\frac{ikt}{2N}\sum_{k<k'}(1+\varepsilon_{kk'})(2\delta_{r_kr_{k'}}-1)\right),\text{where } r_i=s_j,\ r_j=s_i \text{ and } r_k=s_k \text{ if } k\neq i,j.\end{aligned} \tag{10}$$

Here we have used the fact that $s_i\neq s_j$, to identify $\bar{s}_i=s_j$, and $\bar{s}_j=s_i$, which also results in the same $n_\pm$ for the strings labeling c and c^* terms, as they differ only by an interchange of two spins. This also implies that $\sum_{l<l'}\delta_{s_ls_{l'}}=\sum_{l<l'}\delta_{r_lr_{l'}}$, and hence the expression simplifies to

$$\langle O\rangle=\frac{1}{2^N}\sum_{i<j}\sum_{\substack{s_1,s_2,\cdots s_N\in\{+,-\}\\ s_i\neq s_j}}|\alpha_+|^{2n_+(s)}|\alpha_-|^{2n_-(s)}\exp\left(-\frac{2ikt}{2N}\sum_{l<l'}\varepsilon_{ll'}(\delta_{s_ls_{l'}}-\delta_{r_lr_{l'}})\right). \tag{11}$$

For a given pair i,j such that $i<j$, the terms in the exp part are nonzero iff (a) $l=i,\ l'>i, l'\neq j$, (b) $l'=i,\ l<i$, (c) $l=j,\ l'>j$ and (d) $l'=j,\ l<j, l\neq i$. Cases a+b constitute $N-2$ terms while c+d another $N-2$, providing altogether $2(N-2)$ independent ε_{ij} that contribute. In each of these case $\delta_{s_ls_{l'}}-\delta_{r_lr_{l'}}=2\delta_{s_ls_{l'}}-1$. For example in case (a), we have $\delta_{s_ls_{l'}}-\delta_{r_lr_{l'}}=\delta_{s_is_{l'}}-\delta_{r_ir_{l'}}=\delta_{s_is_{l'}}-\delta_{\bar{s}_is_{l'}}$. Therefore only one of the Kronecker terms survive and hence this is same as $2\delta_{s_is_{l'}}-1$. Denoting the set of (l,l') that satisfies any one condition from the four cases above as S_{ij} we have

$$\langle O\rangle=\frac{1}{2^N}\sum_{i<j}\sum_{\substack{s_1,s_2,\cdots s_N\in\{+,-\}\\ s_i\neq s_j}}|\alpha_+|^{2n_+(s)}|\alpha_-|^{2n_-(s)}\prod_{l,l'\in S_{ij}}\exp\left(-\frac{ikt}{N}\varepsilon_{ll'}(2\delta_{s_ls_{l'}}-1)\right). \tag{12}$$

As in each term of the summation the product of exponential terms contain independent $\varepsilon_{ll'}$, performing a disorder averaging with a zero centered Normal distribution yields

$$\begin{aligned}\langle O\rangle_w &= \frac{1}{2^N}\left(\int_{-\infty}^{\infty}\frac{1}{\sqrt{2\pi w^2}}\cos\left(\frac{kt}{N}\varepsilon\right)\exp\left(-\frac{\varepsilon^2}{2w^2}\right)d\varepsilon\right)^{2(N-2)}\sum_{i<j}\sum_{\substack{s_1,s_2,\cdots s_N\in\{+,-\}\\ s_i\neq s_j}}|\alpha_+|^{2n_+(s)}|\alpha_-|^{2n_-(s)}\\ &= \frac{1}{2^N}\left[\exp\left(-\frac{w^2k^2t^2}{2N^2}\right)\right]^{2(N-2)}\sum_{i<j}\sum_{\substack{s_1,s_2,\cdots s_N\in\{+,-\}\\ s_i\neq s_j}}(1+\sin\theta\cos\phi)^{n_+(s)}(1-\sin\theta\cos\phi)^{n_-(s)}\end{aligned} \tag{13}$$

The above summation is constrained, but the i,j positions do not matter any longer and hence there are $N(N-1)/2$ identical smaller sums. For example we may take $s_1=\pm$ and $s_2=\mp$, and we get

$$\langle O\rangle_w=\frac{1}{2^N}\left[\exp\left(-\frac{w^2k^2t^2}{2N^2}\right)\right]^{2(N-2)}N(N-1)\sum_{n_+=1}^{N-1}\binom{N-2}{n_+-1}(1+\sin\theta\cos\phi)^{n_+}(1-\sin\theta\cos\phi)^{N-n_+}, \tag{14}$$

which is easily done using the Binomial theorem to yield

$$\langle O\rangle_w=\frac{N(N-1)}{4}(1-\sin^2\theta\cos^2\phi)\exp\left[-\frac{w^2k^2t^2(N-2)}{N^2}\right] \tag{15}$$

Hence finally we arrive at a compact expression:

$$\begin{aligned}\left\langle J^2(t)\right\rangle_w &= \left\langle J_x^2(t)+J_y^2(t)+J_z^2(t)\right\rangle_w, \\ &= \frac{3N}{4}+\frac{N(N-1)}{4}\left[\sin^2\theta\cos^2\phi+(1-\sin^2\theta\cos^2\phi)\exp\left[-\frac{w^2k^2t^2(N-2)}{N^2}\right]\right].\end{aligned} \tag{16}$$
